# Natural Variations and Dynamic Changes of Nitrogen Indices throughout Growing Seasons for Twenty Tea Plant (*Camellia* *sinensis*) Varieties

**DOI:** 10.3390/plants9101333

**Published:** 2020-10-09

**Authors:** Yange Zhang, Xiangsheng Ye, Xinwan Zhang, Wei Huang, Hua Zhao

**Affiliations:** 1 Key Laboratory of Horticultural Plant Biology of Ministry of Education, Huazhong Agricultural University. Wuhan 430070, China; zhangyg@webmail.hzau.edu.cn (Y.Z.); zhangxinwan@webmail.hzau.edu.cn (X.Z.); huangwtea@webmail.hzau.edu.cn (W.H.); 2 College of Horticulture & Forestry Sciences, Huazhong Agricultural University, Wuhan 430070, China; 3 College of Resources & Environment, Huazhong Agricultural University, Wuhan 430070, China; xiangshengye@mail.hzau.edu.cn

**Keywords:** apparent nitrogen remobilization efficiency, dynamic change, natural variation, nitrogen utilization efficiency, tea plant variety

## Abstract

Tea (*Camellia sinensis* (L.) O. Kuntze) leaves are harvested multiple times annually accompanied by a large amount of nitrogen (N) removed. Therefore, tea plantations are characterized by high requirements of N. This study aimed to assess the variations of N-level, apparent N remobilization efficiency (ANRE), and N utilization efficiency (NUtE) and their dynamic changes during growing seasons for twenty tea varieties. The N-level was highest in the one bud with two leaves as the youngest category, followed by mature leaves attached to green-red stems, and then by aging leaves attached to grey stems. The dynamic N-level presented different profiles of “S”-, “U”-, and “S-like”-shape in the three categories of leaves during the growing seasons. Here, specifically defined ANRE indicated N fluxes in a specific category of leaves, showing that sources and sinks alternate during the period of two consecutive rounds of growth. The dynamic of averaged NUtE followed an “S”-shape. The results revealed annual rhythms and physiological characters related with N indices, which were variety dependent and closely related with the amount of N requirements at proper time. An optimized NUtE is a complex character determined by the combination of tea plantation management and breeding practices to achieve sustainable development with economic benefit.

## 1. Introduction

**Figure A1:**
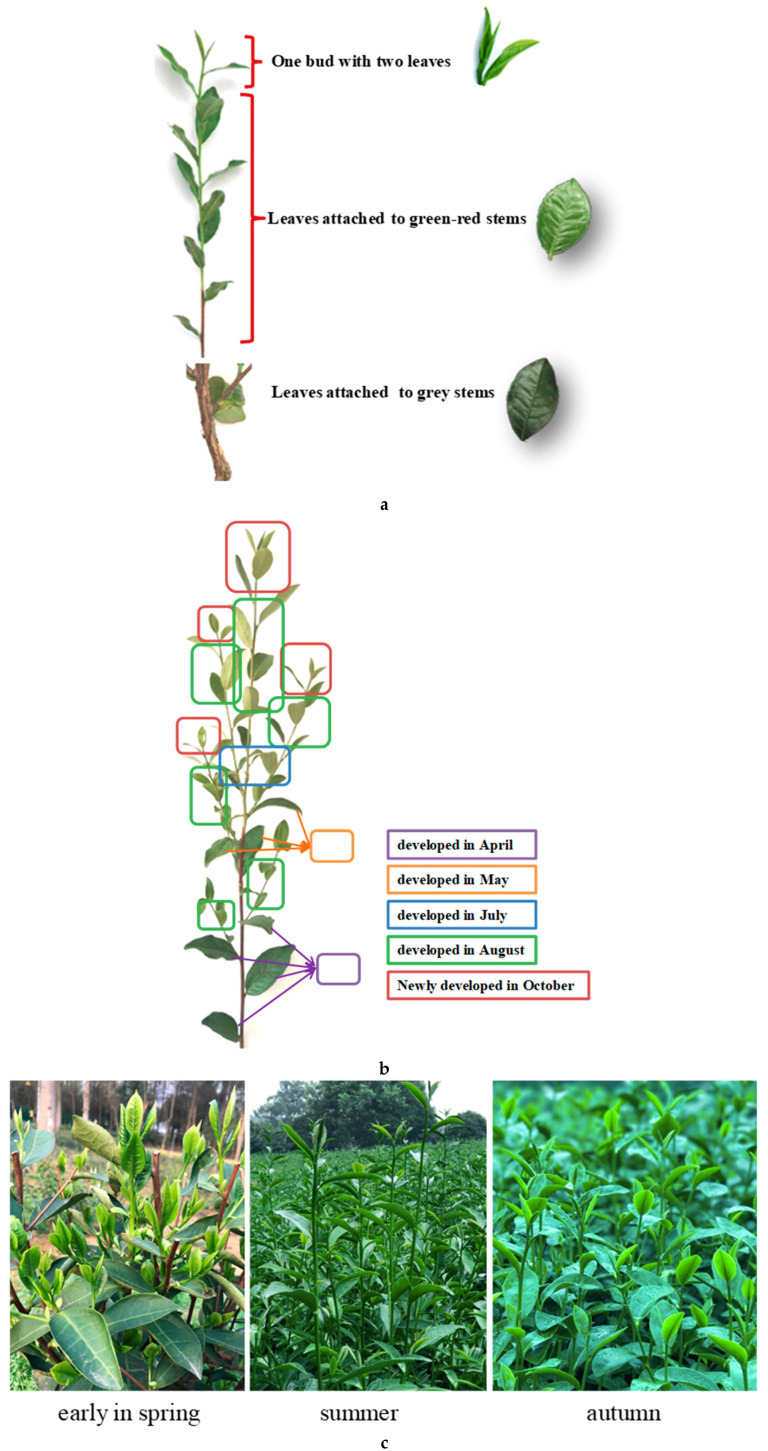
Diagram for tea plant sample standard (**a**); seasonal development of leaves on the tea plant phyllotaxy (**b**); growth performance in a tea garden over the entire season: early spring, summer, and autumn (**c**).

The tea plant (*Camellia sinensis* (L.) O. Kuntze) originated in the southwestern part of China and is a perennial evergreen beverage plant. The aerial portion of tea plant may take on several morphological types: arbor, semiarbor, and shrub, depending on factors such as cultivation practices, environments, and genotypes. Almost all cultivated tea plants showed semiarbor and shrub morphology [1]. Tea fresh young shoots are the harvest part. The phyllotaxy of tea leaves on a stem is alternate. Tea plants have multiple stems of different developmental stages due to the pruning practices. The color of tea plant stems changes from green to red, then to brown, and then to grey brown with age (see Figure A1). The leaves attached to the stems of different color vary in physiological age and distinct function for their physiological status. Considering the standards of plucking for tea making, leaf position on the stems is of great importance: one bud in early spring for famous tea, one bud with one or two leaves for top quality tea, the third or fourth leaf from top for Oolong tea, mature leaves with semilignified stems for dark tea, etc. [2]. Generally, youngest shoots are plucked multiple times annually and leaf yield is highly dependent on tea variety and the tea-making process [2]. Therefore, the tea plant is a unique crop from cultivation to harvest, and breeders worldwide have been focusing on developing tea plant varieties with a better harvest index (HI) to enrich tea products and to improve tea economy [3].

N is an essential nutrient element in most macromolecules and secondary compounds of tea production, such as caffeine and theanine, etc. Caffeine (1,3,7-trimethyl xanthine) is up to 2.80% [4] and theanine (γ-glutamyl-l-ethylamide) is up to 4.53% in young shoots at dry weight [5]. Therefore, N is one of the most important nutrients determining tea quality and yield, and N application is highly related with tea quality as well as economic benefits. Excessive application of N increases the cost of farming, decreases tea quality, and causes soil acidification and consolidation, and it is a serious problem in the region of green tea production [6,7], whereas insufficient application leads to yield and quality reduction. In addition, the severity of soil acidification of tea garden due to overfertilization in tea gardens has gradually become an unpalatable question to achieve high yield; accordingly, it is a contrary to achieving sustainable development [8]. Besides integrated field management techniques or measures, tea yield, quality, and adaptability of tea plant varieties are the priority targets during breeding programs. Moreover, the character of extended growing season, early sprouting, distinguished aroma, and taste characters are receiving more attention [3]. It is undeniable that several practices of cultivation, breeding, and harvest standard could substantially enhance growth vigor and maintain or even increase the harvestable leaves, mainly via enhancing remobilization of prestored N-containing compounds and improved HI, a variable factor particularly in tea production [3,9]. Significant advances have been made in tea breeding concerning understanding of the suitability of tea making categories over the last few decades [3]. However, so far, breeding scientists have not yet focused on nutrient use efficiency of the tea plant; therefore, further increases in yield will necessitate an improvement in nutrient management.

Nitrogen demand is the maximum of the growth activity-driven for crops [10]. As might be expected, the involvements of sophisticated mechanisms for N uptake, translocation, assimilation, and remobilization are important across the plant’s life cycle [11,12,13,14]. Consequently, N use efficiency (NUE, harvest weight/N taken up) and N fertilizer requirement have become imperative to optimize friendly environmental farming [14]. NUE consists of N uptake efficiency (NUpE, N taken up/N supplied) and N utilization efficiency (NUtE, harvested dry weight/N taken up in the harvest), and N supply has been effectively optimized for maximum NUE through breeding [14]. Generally, N supplied by soil is hard to sustain the high N demand for rounds of new bud sprouting and leaf growth, therefore, N remobilization usually occurs within tea plants. The N remobilization is highly affected by nutrient condition within the plant [15] and it is essential at the whole plant level; therefore, the nutrient budget allows the redistribution of N between sources and sinks for growth or adjustment to environmental conditions [16]. For annuals, remobilization of N occurs when the availability of N in soil is insufficient, and then leaf senescence and mature leaves become N sources to support the growth of new organs [17,18]. In woody species, seasonal patterns of N remobilization were observed, and N was remobilized from the trunk to sustain leaf growth each spring [19]. In evergreen trees, N remobilization occurs during vegetative growth synchronously with leaf senescence and shedding [20,21]. For tea, developing tea leaves are the dominant N sinks during the growing seasons, and agricultural practices of pruning and plucking increase sink strength, which inevitably increase the N remobilization. In tea plants, N remobilization associated with vegetative growth during the growth season and dormancy in winter is crucial for tea yield potential and tea quality, as well as related with NUE. The dynamic changes of N level allowed us to trace the amount of N requirement and to determine the N remobilization and redistribution between the leaves of varied tenderness as the two various categories. The bud with two leaves is always the newly developed one for each round of growth, and thus it is the harvest part and there is no point for N remobilization. Many definitions of NUE were summarized [14,22] and were applied to implicate indices related to N budget. The definition of NUE is species specific and it is very difficult for the tea plant because of its perennation, rounds of harvest, and inconsistent harvest index (HI), which depend on the type of tea made. Definition of a specifically appropriate NUE to evaluate tea germplasm is immensely important.

However, the knowledge regarding N dynamic behavior, from uptake to remobilization between source and sink organs, remains largely unknown at the whole level. It is unknown how and how much N is allocated into individual parts in the tea plant, particularly in the genetic variation among tea varieties. Towards gaining insight of these, a better understanding of the processes involved in the elaboration of N partition and remobilization is required. Concerning the importance in N management and NUE, the evaluation of genetic variation among tea varieties is of great significance, and an optimization of apparent N remobilization efficiency (ANRE, apparent ratio of change of N level in a specific category of leaves at different times) is required to improve the NUE. In the present study, the variations of the dynamic NUtE and ANRE are evaluated based on N level during the growing seasons for twenty tea varieties of 10-year-old tea plants.

## 2. Results

### 2.1. Dynamic Changes of N Level in the Three Categories of Leaves during the Growing Seasons

In this study, five rounds of young buds sprouted without plucking for tea making. For the three categories of leaves over the growing period, N level was highest in the youngest leaves and declined as the leaves developed and matured to be the leaves attached to green-red stems. A relatively lower and constant N level was found in the leaves attached to the grey stems.

**Figure 1:**
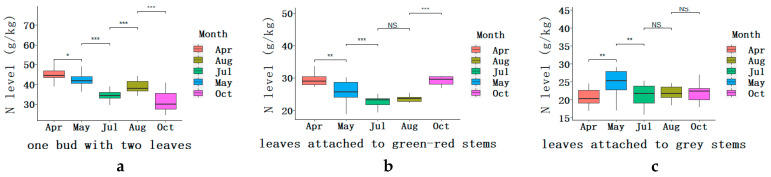
Boxplots for dynamic changes of N level (g N kg^−1^ DW) in the three categories of leaves: one bud with two leaves (**a**), leaves attached to green-red stems (**b**), and to grey stems (**c**), during the growing seasons. The values were calculated based on the twenty tea varieties described in Table A1. The box represents the interquartile range (IQR), the bisecting line represents the median, and the whiskers represent 1.5 times the IQR. Asterisks indicate significant differences between averaged N levels of two consecutive rounds, * *p* < 0.1, ** *p* < 0.05, *** *p* < 0.01 (*t*-test); NS, not significant.

**Figure A2:**
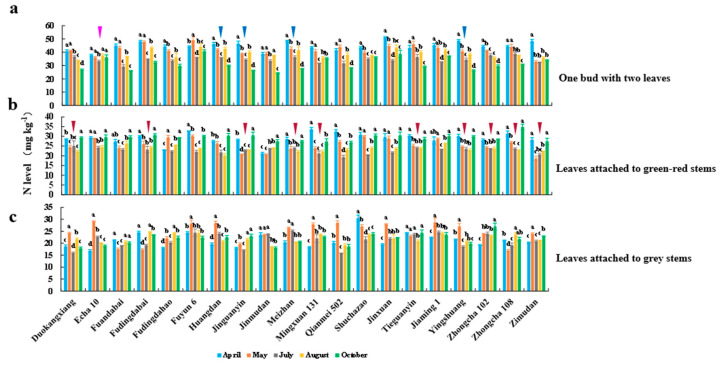
Dynamic changes of N level (g N kg^−1^ DW) in the three categories of leaves, one bud with two leaves (**a**), leaves attached to green-red stems (**b**), and to grey stems (**c**) during the growing seasons. The blue triangles represent the four varieties showing the same pattern of dynamic change of N level of one bud with two leaves throughout the growing season, and the pink triangle shows a specific pattern in Echa 10. The red triangles indicate the ten varieties showing the same pattern of dynamic change of N level in the leaves attached to green-red stems throughout the growing season. Values are means ± standard (SD) (*n* = 3). Error bars indicate SD. Significant differences are indicated by letters a, b, c, d, and e (*p* = 0.05).

The dynamic changes of N level for one bud with two leaves were highly variable between two consecutive rounds during the growing period. The average N level decreased from 44.2 g kg^−1^ early in April to 41.55 g kg^−1^ in May, to 34.50 g kg^−1^ in July, and followed by a transient increase up to 39.0 g kg^−1^ in August, and then decreased thereafter with the lowest of 31.05 g kg^−1^ in October, being an S-shaped trend (Figure 1a). Natural variations of N level among the twenty elite tea plant varieties were indicated by coefficient of variation (CV), 15.16% (October) > 9.43% (May) > 8.32% (April) > 8.07% (July) > 7.08 (August) (Table 1), showing that the variation among the tea varieties was relatively limited in spring and extensive in autumn. As expected, significant differences of N level were observed between rounds of samples over the growing period. For individual tea varieties, similar seasonal patterns of N level were observed for four tea varieties, Huangdan, Jinguanyin, Meizhan, and Yingshuang. The N levels were significantly higher in April than those in May and August, and followed by July, and lowest in October (Figure A2). Echa10 showed a relatively limited range of N level, and it was a bit significantly lower than four other rounds (Figure A2).

The averaged N level for leaves attached to green-red stems decreased from April’s 27.99 g kg^−1^ to July’s 22.67 g kg^−1^, whereas it increased to 23.62 g kg^−1^ in August and then to 29.42 g kg^−1^ in October, being a U-shape trend (Figure 1b). The variation of N level among the 20 tea varieties was indicated by CV, presenting relatively high value of 13.17% in April and 12.32% in May, followed by 6.08% in October, 6.06% in July and 5.65% in August (Table 1). The results showed different patterns of N level variation among the tea varieties: extensive in spring season and limited in summer season. It was intriguing that almost half of the tea varieties presented similar profiles of N levels, indicated by triangles in Figure A2, showing significantly lower values in the consecutive samples of May, July, and August, compared with those of April and October (Figure A2).

Concerning the average N level in the leaves attached to grey stems, it increased from 20.79 g kg^−1^ in April to 24.57 g kg^−1^ in May at a peak level, and then decreased to 21.19 g kg^−1^ in July, and remained relatively constant, 21.93 g kg^−1^ in August and 21.71 g kg^−1^ in October. It followed an S-like shape throughout the growing seasons, suggesting it reached a plateau during the summer and autumn seasons. Overall, the relatively low N level in the leaves attached to grey stems was remained constant over the entire growing period. A more specific phrase differed significantly in N level among the 20 elite tea plant varieties with CV of 16.60% (April) > 16.16% (May) > 13.60% (July) > 11.11% (October) > 8.16% (August) (Table 1), which showed an obviously descending trend from April to August, and a reversely increasing trend from August to October. Similarly, significant variations between rounds of leaves attached to grey stems were found in each tea variety (Figure A2).

### 2.2. Overall Comparison of N Level between the Three Different Categories of Leaves

During all the growing seasons, N level was highest in the one bud with two leaves, followed by the leaves attached to green-red stems, and then the leaves attached to grey stems. The differences of N level between one bud with two leaves and the leaves attached to green-red stems were particularly obvious during the period of most rapid growth, in spring (April and May) and summer (July and August); however, it was not the case in autumn (October). Notably, distinctive differences of N level between the leaves attached to green-red and to grey stems were observed in early spring (April) and autumn (October), whereas there was no obvious difference during the vigorous growing period, from May to August. As mentioned above, dynamic changes of N level during all the growing seasons differed significantly among the three categories of leaves, and it showed a largest fluctuation in the bud with two leaves, followed by the leaves attached to green-red stems, and then by the leaves attached to grey stems.

Interestingly, different categories of leaves exhibited a peak of N level during all the growing seasons, and peaks appeared in May for most genotypes in the leaves attached to the grey stems (Figure 1c). For the two other categories, they exhibited similar patterns over the growing seasons between April and July, from an average peak level to a consecutive downward trend. Over the following growing seasons, from July to October, the two categories showed distinctive trends, showing gradual increase at a peak level in August first and thereafter decline in the bud with two leaves. However, for the leaves attached to green-red stems, there was a weak decline first and it was maintained at a low level for the period from July to August, followed by a substantial increase from August to October (Figure 1b), which was nearly equal to those in spring. The fluctuations of N level in the three categories of leaves presumably indicated the dynamic changes of N demand concomitant with round-by-round of bud sprout.

### 2.3. Dynamic Changes of Apparent N Remobilization Efficiency in Different Leaves during the Growing Seasons

**Figure 2:**
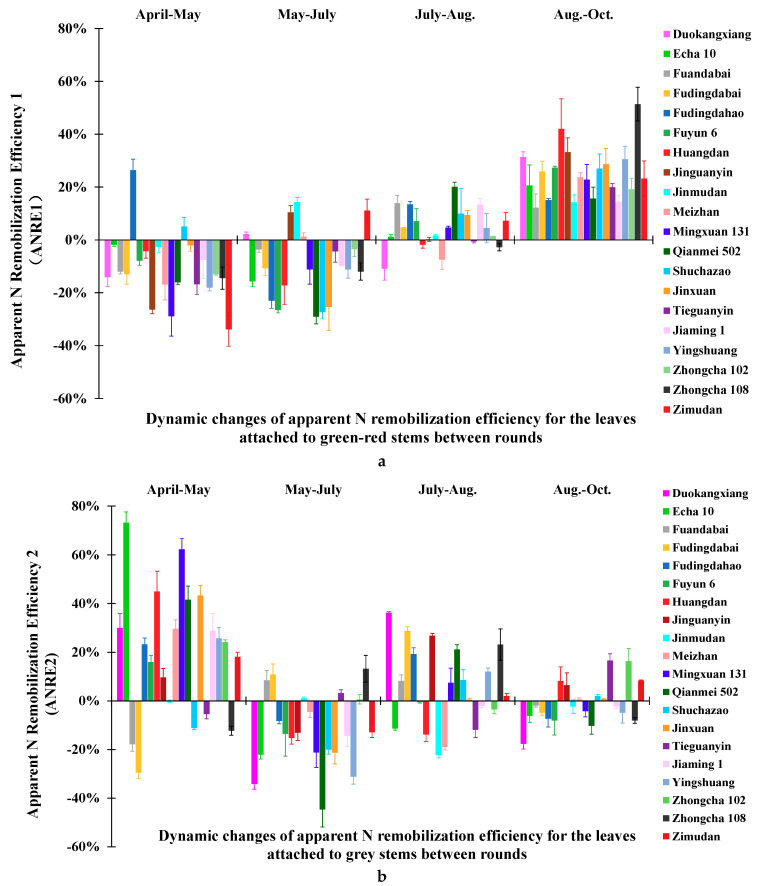
Dynamic changes of apparent N remobilization efficiency for leaves attached to green-red stems (**a**) and to grey stems (**b**) of the twenty tea plant varieties during the growing seasons. Values are means ± standard (SD) (*n* = 3). Error bars indicate SD.

The dynamic changes of ANRE of the twenty tea varieties are presented for the two categories of canopy leaves, ANRE1 and ANRE2, for the leaves attached to green-red (Figure 2a) and to grey stems, respectively (Figure 2b). For the leaves attached to green-red stems, during the period from April to May, ANRE1 < 0 was presented in eighteen varieties, ranging between −33.86% and −2.12% (Figure 2a). By contrast, two other varieties showed contrasting ANRE1, ANRE1 > 0, being 5.06% for cv. Shuchazao and 26.39% for cv. Fudingdahao. During the next period between the second and third round, a series of fifteen varieties showed ANRE1 < 0 that varied between −29.11% and −3.51%, and another five varieties were ANRE1 > 0, ranging between 1.24% and 14.30%. For the third period between the third and fourth round, from July to August, conversely, fifteen varieties showed ANREs > 0 and five varieties ANRE1 < 0. The range for ANRE1 > 0 was moderately limited, from 0.19% to 20.14%. The scope for ANRE1 < 0 was further narrowed, from −10.94% to −0.63%. During the fourth period between the fourth and fifth round, from August to October, all varieties exhibited ANRE1 > 0 with an obviously extensive range between 12.21% and 51.35%.

For the leaves attached to grey stems, they were almost one year old and approached senescence. During the first period between the first two rounds, from April to May, only five varieties presented ANRE2 < 0, ranging between −29.56% and −0.15%. By contrast, fifteen other varieties showed ANRE2 > 0 and ranged between 9.61% and 73.15%. During the second period between the second and third round (from May to July), a total of fifteen varieties were ANRE2 < 0 and varied between −44.73% and −4.50%, and five other varieties were ANRE2 > 0, ranging between 0.64% and 13.16%. Compared with those of the first period, the interval of ANRE2 in the second period was greatly reduced for ANRE2 > 0, whereas it was greater for ANRE2 < 0. During the third period between the third and fourth round (from July to August), the result showed that twelve varieties were ANRE2 > 0, varying from −0.48% to −22.33%, and eight others were ANRE2 < 0, varying between 0.51% and 36.23%. During the fourth period between the fourth and fifth round (from August to October), twelve varieties exhibited ANRE2 < 0 with a range between −1.95% and −17.69%, and the range for ANRE2 > 0 was approximately comparable to that of ANRE2 < 0, ranging from 0.61% to 16.53%. These alternating changes of ANRE2 raise a possibility that N remobilization could be more likely induced by a new round of bud growth rather than leaf senescence.

Interestingly, a comparison of ANRE between the two categories of canopy leaves showed an almost contrary pattern in April–May, except for Fudingdahao being both ANRE1 > 0 and ANRE2 > 0. For the period of May–July, nine tea varieties showed converse trends in the both canopy leaves, five tea varieties presenting ANRE1 < 0 and ANRE2 > 0, and four varieties presenting reverse patterns.

Overall, the ANRE in the two categories of leaves varied significantly among tea varieties, which was indicative of N dynamic behavior within tea plants. During April–May, most tea varieties showed ANRE1 < 0, implying the leaves attached to green-red stems played a role of source organ, and contrarily, the leaves attached to grey stems of most tea varieties were supposed to act as a sink organ with ANRE2 > 0. The difference of growth rhythm and N capacity among the tea varieties was probably responsible for the discrepancy of the two tea varieties, Fudingdahao and Shuchazao in the leaves attached to green-red stems (Figure 2a) and five tea varieties in the leaves attached to grey stems (Figure 2b). During May–July, the finding that ANRE1 < 0 in the fifteen tea varieties and ANRE2 < 0 in the fourteen tea varieties suggested the function as source leaves of the two categories of leaves. During July–August, it is interesting that most tea varieties showed ANRE > 0 in the two categories of leaves and thus the two categories of leaves of the tea varieties probably played the role of sink organs. During August–October, all tea varieties showed ANRE1 > 0 with relatively high values, and twelve tea varieties showing ANRE2 < 0 and eight showing ANRE2 > 0 were predicted as source and sink leaves, and the capacities were greatly reduced compared with those leaves attached to green-red stems. It is hard topredict whether or not there is “luxury-N”, although the value of ANRE is extremely low, either > 0 or < 0.

### 2.4. Dynamic Changes of N Utilization Efficiency (NUtE) during the Growing Seasons

**Figure 3:**
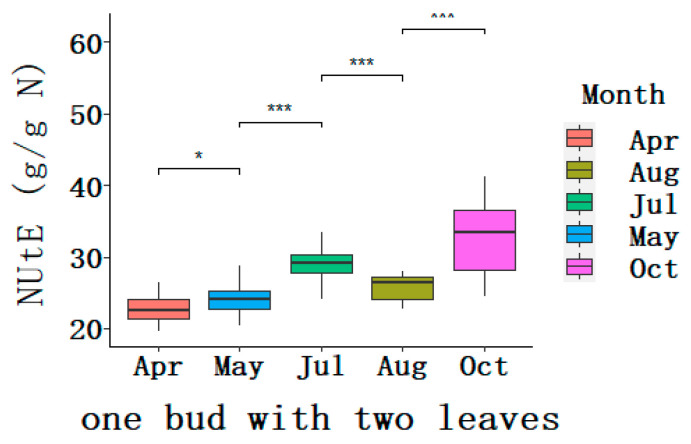
Boxplot for dynamic N utilization efficiency (NUtE, g/g N) for the harvest leaves of the twenty tea plant varieties during the growing seasons. The box represents the interquartile range (IQR), the bisecting line represents the median, and the whiskers represent 1.5 times the IQR. Asterisks indicate significant differences between two consecutive averaged NUtEs, * *p* < 0.1, *** *p* < 0.01 (*t*-test).

The N assimilation efficiency and remobilization efficiency are related with NUtE in the tea plant. Generally, the part of one bud with two leaves is harvested for tea making and NUtE was specifically calculated for it. The averaged NUtE from April to July increased from 22.76 to 29.05 g g^−1^ N, and decreased to 25.77 g g^−1^ N in August, and then conversely increased up to 32.99 g g^−1^ N in October; the dynamic change of NUtE followed an S-shape (Figure 3). Among the tea varieties tested, the NUtE ranged between 19.51 and 26.39 g g^−1^ N in April, and between 22.70 and 27.94 g g^−1^ N in August, which was in a relatively low and limited range and indicated that N was in great demand in the youngest part for harvest. The values of NUtE were 20.44−30.35 g g^−1^ N in May and 24.09−34.15 g g^−1^ N in July, which were significantly extended. Particularly in October, at 24.53–41.25 g g^−1^ N, the spectrum was further enlarged. Natural variations of NUtE among the twenty tea plant varieties were indicated by coefficient of variation (CV), 14.76% (October) > 10.18% (May) > 8.73% (April) > 8.17% (July) > 7.02 (August) (Table 2), which showed the most extensive variation in autumn and relatively limited variation in spring and summer, which were similar with those of the coefficient of variation for the N level.

The obviously dynamic NUtEs were observed during the growing period for each tea plant variety and significant variations were found among the twenty tea plant varieties (Table A2). Interestingly, the ranges of NUtE of five varieties: cv. Echa10, cv. Huangdan, cv. Shuchazao, cv. Jinxuan, and cv. Jiaming 1, were extremely limited, showing a very consistent pattern with a peak in July and valley in April. By contrast, in the two tea varieties, the NUtE showed a broad range and differed almost two fold between the maximum in October and minimum in April, 20.54−41.25 g g^−1^ N for cv. Meizhan and 20.35–38.05 g g^−1^ N for cv. Yingshuang. For the remaining tea plant varieties, trends of NUtE went upward from April to July, reversed and went downward between July and August, and finally went upward and peaked in October.

## 3. Discussion

The relationships between tea yield and quality, and ANRE and NUE are complex and remain unknown. To this end, an integrative evaluation for N indices is of great significance to reveal N behavior within tea plants. Furthermore, it is particularly essential and necessary to figure out the genotypic variations and dynamic changes over all the growing seasons, how these traits contribute to NUE, and how they interact with nutrient management practices. Proper N management increases growth rate during rounds of tea bud growth and enhanced N remobilization usually leads to a higher HI. In addition, considerable remobilization of N toward sinks might improve NUE, thereby tea quality is improved due to an increased level of amino acid as a leaf usage crop; however, the requirement of N fertilizer is difficult to predict. Therefore, it is necessary to increase the sink capacity to increase NUE; of course, it should be balanced by source N remobilization.

According to the seasonal changes of N level in the youngest leaves, similarly dynamic changes between these varieties probably indicates that they share identical rhythm of sprout initiation and growth rate. For the leaves attached to green-red stems between May and August, ten varieties showed similarly seasonal changes of lower and comparable N levels, suggesting possible roles of source organ and sink organ in the periods of April–May and August–October, respectively. Over the entire growing period, a relatively low and constant N level in leaves attached to grey stems probably suggests their low capacity as sinks or sources, compared with the leaves attached to grey stems.

In perennial switchgrass, the correlation between time interval from heading to harvest and ANRE, as well N level in senescent tillers, was proved [23]. Thus, it indicated the practicability of the ANRE formula based on N level in this study. N requirement is the maximum of the growth activity-driven demand for crops [10]. N application at different stages or time is a possibility to increase NUE because N sink capacity is regulated by developmental stage, which is variety dependent [24]. The ANRE shows great promise in terms of an effective management technique in increasing fertilizer NUE. It was exhibited by reducing N fertilizer by 32% and increasing grain yield by 5% in a site-specific fertilizer experiment [25]. Here, N application at a proper time was indicated and they were tea variety dependent by their patterns of seasonal changes of N level and derived ANRE. Nitrogen was remobilized from both categories of canopy leaves at different levels of efficiency among the twenty tea varieties. ANRE highlights the role of apparent N remobilization with the rounds of bud sprout and it is critical to improve NUE. ANRE showed how N participated in the two categories of tea leaves. Theoretically, when ANRE < 0 in a specific period, N appeared to be exported from a specific category of leaves as source organs, suggesting the amount of N taken up by the root was insufficient to meet the requirement of growth, thereby N was remobilized outward and reused within the plant to improve the NUE consequently. Conversely, when ANRE > 0 in a period, N was transported inward to the specific leaves as N sink organs to accumulate N. Therefore, the capacity difference of N between the categories of leaves would depend on the net amount of N taken up from the soil by the roots for the requirement of new buds. To take specific varieties, four combinations of ANRE1 and ANRE2 were found in all the tea varieties. First, the cases of ANRE1 > 0 and ANRE2 > 0 suggested that two categories of leaves function as N sinks, suggesting a significant amount of N is required to be taken up by roots in this period, and that N application ahead is exactly suitable and necessary, as shown by the Fudingdahao variety in the period of April–May (Figure 2). Conversely, the varieties of ANRE1 < 0 and ANRE2 < 0 suggested two categories of leaves function as N sources, implying a strong sink strength exists because of new buds’ sprout or rapid growth, therefore N supplement is necessary immediately after this period, such as shown by the Fuandabai, Fudingdabai, Tieguanyin, Zhongcha 108, etc. varieties in the period of April–May (Figure 2). Therefore, it can be concluded whether the N amount taken up by the root is enough for the growth of new buds according to the ANRE of the two categories of leaves attached to green-red stems and to grey stems. The tea varieties of ANRE1 > 0 and ANRE2 < 0 suggested leaves attached to green-red stems and to grey stems function as sinks and sources, respectively, such as Duokangxiang in August–October, and on the contrary, leaves attached to green-red stems and to grey stems functions as sources and sinks, respectively, as in Duokangxiang for July–August. For the latter two cases, the amount and timing of N application depends on the specific situation of the capacity difference between sinks and sources.

From an agronomical perspective, the timing and efficiency of N remobilization are immensely important, not only for reducing excess N application and improving NUE, but relating to yield potential and tea quality [26]. In agricultural tea production, the pattern of a significant amount of N fertilizer applied late in autumn as basal and small part early in the spring before bud sprouting as topdressing fertilization, is an established farming practice and popular in most tea areas of China, regardless of the difference of N requirements for individual tea plant varieties. This inevitably leads to substantial amounts of N loss through gaseous N emissions, soil denitrification and volatilization, surface leaching, and severe environmental hazards [27,28,29].

In recent years, the technique of formula fertilization by soil testing was widely popularized in tea-planted areas [30]. Therefore, reasonable definition for nutrient demand and partition is a prerequisite. This is particularly critical to compare varieties with regard to adaptability of making different tea types. Despite of the shortage of leaf yield and derived N allocation, these results highlighted a crucial role of N level to indicate N requirement during the growing seasons. Thus, optimized fertilization is of great importance to reduce input and increase NUE, and it is both an economically and environmentally desirable measure. Somewhat surprisingly, there were obvious regularities in the dynamic changes for N level, which proved the validity and suitability of the leaf sample at the specific developmental stages to investigate seasonal N demand.

N remobilization is strongly dependent on genotypes in multiple crops [31,32,33,34], therefore it is particularly critical when comparing varieties adaptable for making different tea types. Once a new round of buds sprout and leaves develop, N is remobilized from the reserves to the youngest buds as sinks to meet their requirements. This remobilization is supposed to act as sink activity [35] to stimulate senescence of leaves attached to grey stems. If N requirements of harvest part are not met due to ineffective N budget within intrashoots, N levels in harvest leaves decline, which would subsequently lead to a decline in tea quality. Therefore, ANRE is another candidate parameter for N management or NUE. Remobilization of N taken up previously plays an important role throughout plant growth; surprisingly, breeding can alter N in the sink organs where it is sourced. For instance in maize, grain N source has changed via breeding over time and modern maize varieties, during the reproductive phase, take up N directly and transport more into the grain [36]. Understanding the N sources in the harvestable parts, either from remobilization within plants or from uptake, is of significant importance to help guide future improvements in yield and NUE of crops. Therefore, there are several plausible explanations for the relatively low ANRE: a lack of sink strength, source/sink ratio, or adequate supply taken up from soil solution. The discrepancy of ANRE among the twenty tea varieties might be due to the inconsistency of tea bud sprouts or growth rate, which are probably genetically controlled properties. Therefore, the comparison between varieties of ANRE is suitable and meaningful for those showing similar growth rhythm. For economic and ecological considerations, the high crop-specific N balance surplus has to be reduced without reducing the current yield and quality. The suitability of tea making of tea varieties is mainly determined by the free amino acid level, the ratio of polyphenols to amino acids, and flavor characteristics, etc. [3]. In this study, the finding showed a possible connection between the profile of ANREs and growth rhythm, rather than between the profile of ANREs and suitability of tea making. Probably, similar genetic background is responsible for the N indices and N application features.

The characteristics of tea plant variety response to N, how they are regulated by field management practices, and how they contribute to NUE, remain unknown. A promising approach is to breed N-efficient varieties, thereby increasing yield under limited N and potential yield under normal N conditions, which thus allows for decreasing the requirement of N application to achieve optimum yields and maintain quality [37,38]. Plant NUE is a complex trait that is governed by many physiological processes, including N uptake, assimilation, transport, allocation, and remobilization, as well as the environmental factor of soil N availability. Increases in NUE have been achieved by breeding, cultivation, and genetic improvement approaches by overexpression of key genes involved in N uptake, assimilation, and transport [39,40,41,42]. Considering success in NUE improvement, natural variations and quantitative genetic approaches are definitely worth exploring in tea plants.

## 4. Materials and Methods

### 4.1. Plant Materials and Growth Conditions

The experimental site is located at Huazhong Agricultural University in Wuhan (30°32.87′ N, 114°25.01′ E, 28 m), China. It is an area with a warm and subtropical climate suitable for tea plantation, an average annual temperature of 15.8–17.5 °C, average annual precipitation of 1269 mm, average annual 270 days frost-free period in the past three decades. Spray irrigation was used as a supplement when watering is required. Before basal fertilizer application in 2016, the soil was characterized by a low pH level (about 4.5), organic matter of 42.65 g kg^−1^, alkali-hydrolyzable N of 164.83 mg kg^−1^, available P of 68.06 mg kg^−1^, and available K of 210.36 mg kg^−1^ in the soil layer from 0 to 20 cm depth, which indicated high fertility soil for tea plant plantation. Totally, twenty ten-year-old clonal tea plant (*Camellia sinensis* L.) varieties (described in Table A1) were originally cultivated in 2005 according to the standards of agricultural production. The tea garden has been managed as an integrated annual fertilization practice, once for basal fertilizer in autumn (between mid October and mid November) and twice for topdressing fertilizer (before sprouting early in March and immediately after spring tea harvest) during growing seasons. N was applied with the ratio of 7:2:1 for the three times at the rate of 600 kg N ha^−1^, a relatively moderate N level to achieve high yield. Phosphorus (as P_2_O_5_) and potassium (as K_2_O with SO_4_^2−^ type) were applied with a ratio of N:P_2_O_5_:K_2_O = 2:1:1 as basal fertilizer in autumn. Insects, diseases, and weeds were controlled by chemical or manual methods to ensure vigorous growth and considerable yield. These results suggested that tea plants grew with adequate nutrient levels with standardized culture conditions.

### 4.2. Sampling and N Level Measurements

To highlight the biological significance, the canopy leaves were separated into three categories along the vertical canopy layer: one bud with two leaves (youngest), leaves attached to green-red stems (developed mature leaves), and leaves attached to grey stems (aging leaves) (Figure A1). For each tea variety, the three categories of leaves were collected from early in April to mid October in 2017. Tea plants at the end of each tea row were not considered to avoid border effects.

It is difficult to determine the N amount retained in the lignified stems and roots of tea because it is perennial. Dynamic N levels of the three categories of leaves were quantitatively determined for twenty tea varieties over the growing seasons, starting spring tea in early April and ending autumn tea in October before pruning. Five rounds of sprout buds were as follows: two rounds of spring tea in April and May, two rounds of summer tea in July and August, and one of autumn tea in October. Generally, the category of one bud with two leaves functions as an N sink, and then grow to physiological maturity, as leaves attached to the green-red stems. The leaves attached to the grey stem mostly developed in the previous year.

The sampled leaves were treated at 120 °C for 10 min, dried at 65 °C to constant weight, and then ground to a uniform fine powder by a plant mill. The digestion for samples and determination of N level using a flow injection analysis instrument (a FIAstar 5000 analyzer, FOSS, Hilleroed, Denmark) are described in Yu et al. [43].

### 4.3. Definitions of Apparent N Remobilization Efficiency (ANRE) and N Utilization Efficiency (NUtE)

To estimate apparent N remobilization in the three distinctive categories of leaves during the growing seasons, the ANREs were calculated by dividing N level difference between the B^th^ and A^th^ round divided by that of the A^th^ round. Therefore, apparent N remobilization efficiency (ANRE) was defined for the two categories of canopy leaves as ANRE = (C_B_−C_A_)/C_A_, and C_A_(C_B_) is the N level of the A^th^(B^th^) round specific for the leaves attached to green-red stems or to grey stems. ANRE1 and ANRE2 were special for the canopy leaves attached to green-red stems and grey stems, respectively. Therefore, four groups of ANREs were calculated between the five rounds for the two categories of leaves, which were used to obtain a dynamic view of internal remobilization of N during the growing seasons. ANRE is assumed as one of the most responsive traits as it integrates the N level of two consecutive growth rounds into a single trait.

NUtE is the leaf yield based on the N content involved, and it is therefore considered as a preference to optimally characterize N efficiency especially for tea plant; additionally, overall NUtE is an integration of all harvested leaves round-by-round throughout the growing periods within a year. Therefore, NUtE is calculated by the dry weight divided by the N within this part, NUtE = DW/NC, where DW and NC are dry weight and nitrogen content in the harvested part, respectively. The value of NUtE is the reciprocal of the N level in the harvested part.

### 4.4. Data Analysis

Relevant boxplots were made using the R project ggplot2 package [44]. The significance of a difference was performed using the ggsignif package for the groupwise comparisons. Measurement data were compared with a *t*-test.

## 5. Conclusions

We showed the dynamic changes and natural variations of N indices for twenty ten-year-old clonal tea varieties throughout growing seasons, including the N levels in the three categories of leaves, ANREs in the canopy leaves, and NUtE in the young bud leaves. Despite the variations among varieties, specifically obvious trends were observed for the dynamic changes of N level, S, U and S-like shapes for the three categories of leaves over all the growing seasons. The result revealed an annual growth rhythm and physiological characteristics related to N indices, which were variety dependent. For bud sprouting and growth round-by-round, definitions of ANRE and NUtE were described here specifically for tea. According to the dynamic ANREs, the extent of N remobilization from source to sink/young developed leaves varied significantly among the tea plant varieties, which is indicative of N dynamic behavior within the tea plant. Highly related with tea quality, NUtE for the harvest shoots never should be the most important, and usually NUE is improved especially under less optimal N conditions but with loss of tea yield and quality.

To achieve sustainable development, future work should focus on determining nutrient status for the soil, nutrient demand for tea plant varieties, and management practice attributes that contribute the most to tea production with different tea products: organic tea and conventional pollution-free tea, top-quality tea, and common tea.

## Figures and Tables

**Table 1:** Summary for N levels (g kg^−1^) in the three categories of leaves for the twenty tea varieties over the entire growing seasons.

	April	May	July	August	October
	N level in one bud with two leaves				
Maximum	51.25 ± 0.64	48.98 ± 0.17	41.52 ± 0.39	44.12 ± 0.10	40.78 ± 1.10
Minimum	37.90 ± 0.79	32.97 ± 0.25	29.32 ± 1.36	35.79 ± 0.86	24.24 ± 0.50
Mean	44.2	41.55	34.50	39.0	31.05
Standard Deviation (SD)	3.68	3.91	2.78	2.70	4.70
Coefficient of Variation (CV, %)	8.32%	9.43%	8.07%	7.08%	15.16%
	**N level in the leaves attached to green-red stems**				
Maximum	32.65 ± 2.24	30.06 ± 0.67	25.02 ± 1.69	26.37 ± 1.18	34.80 ± 0.09
Minimum	17.32 ± 1.13	18.64 ± 0.52	19.31 ± 0.96	19.31 ± 0.11	26.83 ± 0.62
Mean	27.99	25.47	22.67	23.62	29.42
Standard Deviation (SD)	3.68	3.13	1.37	1.33	1.79
Coefficient of Variation (CV, %)	13.17%	12.32%	6.06%	5.65%	6.08%
	**N level in the leaves attached to grey stems**				
Maximum	30.46 ± 0.27	29.08 ± 0.27	25.23 ± 0.31	24.65 ± 0.28	26.94 ± 1.46
Minimum	13.76 ± 1.70	16.93 ± 0.39	15.75 ± 0.20	18.47 ± 0.14	17.12 ± 0.62
Mean	20.79	24.57	21.19	21.93	21.71
Standard Deviation (SD)	3.45	3.97	2.88	1.79	2.41
Coefficient of Variation (CV, %)	16.60%	16.16%	13.60%	8.16%	11.11%

**Table 2:** Summary for NUtE (g/g N) in the part of one bud and two leaves for the twenty tea varieties over all the growing seasons.

	April	May	July	August	October
Maximum	26.38	30.33	34.10	27.94	41.24
Minimum	19.51	20.41	24.08	22.66	24.52
Mean	22.74	24.29	29.17	25.75	32.91
Standard Deviation (SD)	1.99	2.47	2.38	1.81	4.86
Coefficient of Variation (CV, %)	8.73%	10.18%	8.17%	7.02%	14.76%

## Appendix A

**Table A1:** The background information for the twenty tea varieties for trait determination related to nitrogen.

Codes	Varieties	Breeding Strategy	Genetic Background	Time for First Harvest	Processing Suitability for Tea Categories
**1**	Duokangxiang	pedigree selection breeding	Selection from landraces population, Yuexi county, Anhui province	Early in April	Green tea
**2**	Echa 10	Pedigree selection breeding	Selection from Taizicha population	Early in April	Green tea
**3**	Fuandabai	Pedigree selection breeding	Selection from Fuandabai population	Late in March/ Early in April	Green/Black/White tea
**4**	Fudingdabai	Pedigree selection breeding	Selection from Fuding Dabaicha population	Late in March/ Early in April	Green/Black/White tea
**5**	Fudingdahao	Pedigree selection breeding	Selection from landraces of Fuding population, Fuding county, Fujian province	Late in March/ Early in April	Green/Black/White tea
**6**	Fuyun 6	Cross breeding	cv. Fudingdabai× cv. Yunnandaye	Late in March	Green/Black/White tea
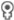
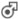
**7**	Huangdan	Pedigree selection breeding	Selection from local population	Late in March	Oolong/Green/Black tea
**8**	Jinguanyin	Cross breeding	cv. Tie guanyin× cv. Huangdan	Late in March	Oolong/Green tea
**9**	Jinmudan	Cross breeding	cv. Tie guanyin× cv. Huangdan	Late in March	Oolong/Green/Black tea
**10**	Meizhan	Pedigree selection breeding	Selection from Meizhan population	Late in March/ Early in April	Oolong/Green/Black tea
**11**	Mingxuan131	Pedigree selection breeding	Selection from Mingshan population	Middle in March	Green tea
**12**	Qianmei 502	Cross breeding	cv. Fengqing dayecha× cv. Xuan’en Changye	Early in April	Black tea
**13**	Shuchazao	Pedigree selection breeding	Selection from landraces population, Shucheng county, Anhui province	Early in April	Green tea
**14**	Jinxuan	Cross breeding	cv. Tainong 8× cv. Yingzhihongxin	Early in April	Oolong tea
**15**	Tieguanyin	Pedigree selection breeding	Core parent for oolong tea, selected from Anxi county, Fujian province	Late in March	Oolong/Green tea
**16**	Jiaming 1	Pedigree selection breeding	Selection from Wuniuzao population	Early in March	Green tea
**17**	Yingshuang	Cross breeding	cv. Fudingdabai× cv. Yunnandaye	Early in April	Green/Black tea
**18**	Zhongcha 102	Pedigree selection breeding	Selection from Longjing population	Early in April	Green tea
**19**	Zhongcha 108	Mutation breeding	Radiation mutation pedigree from cv. Longjing 43	Middle of March	Green tea
**20**	Zimudan	Pedigree selection breeding	An hybrid line selected from natural crossing with cv. Tie guanyin as female parent	Late in March	Oolong tea

**Table A2:** NUtE (g/g N) in the part of one bud with two leaves for the twenty tea varieties over the entire growing seasons.

	Varieties	April	May	July	August	October
**1**	Duokangxiang	24.51	24.18	27.17	27.21	37.13
**2**	Echa 10	26.38	27.69	30.09	26.72	27.59
**3**	Fuandabai	22.25	23.07	34.10	27.29	38.46
**4**	Fudingdabai	20.75	20.92	28.84	23.10	32.97
**5**	Fudingdahao	22.57	24.33	29.76	27.50	33.53
**6**	Fuyun 6	22.48	20.41	27.82	22.66	24.52
**7**	Huangdan	21.62	23.78	28.03	23.42	33.46
**8**	Jinguanyin	21.50	25.69	29.08	24.95	38.20
**9**	Jinmudan	25.73	25.53	29.81	26.60	41.24
**10**	Meizhan	20.54	23.56	27.47	24.00	36.32
**11**	Mingxuan 131	22.89	24.58	31.50	27.89	28.37
**12**	Qianmei 502	24.12	23.91	31.45	26.32	35.63
**13**	Shuchazao	22.95	25.15	28.58	26.77	27.69
**14**	Jinxuan	19.51	22.38	29.51	22.92	25.77
**15**	Tieguanyin	23.13	21.72	27.38	24.97	36.33
**16**	Jiaming 1	22.08	28.67	30.62	24.20	27.04
**17**	Yingshuang	20.52	24.59	29.11	25.93	38.04
**18**	Zhongcha 102	26.38	22.58	24.08	27.94	33.85
**19**	Zhongcha 108	24.21	22.70	25.69	26.63	32.66
**20**	Zimudan	20.65	30.33	33.35	27.90	29.45
